# Delta opioid receptors: Overlooked outlier or the next big thing

**DOI:** 10.1016/j.coph.2025.102528

**Published:** 2025-05-08

**Authors:** Amal El Daibani, Gwendolyn Burgess, Lauren Rysztak, Emily Jutkiewicz, Amynah A. Pradhan

**Affiliations:** 1 Center for Clinical Pharmacology, Department of Anesthesiology, Washington University in St. Louis, St. Louis, MO, USA; 2 Department of Pharmacology, University of Michigan, Ann Arbor, MI, USA

## Abstract

The delta opioid receptor (DOR) is an often-overlooked member of the opioid receptor family, lacking the euphoric and addictive effects of mu receptors and the dysphoric properties of kappa. Instead, the DOR functions to restore balance, showing minimal impact in acute pain but alleviating allodynia and hyperalgesia associated with chronic pain and producing anxiolytic and antidepressant-like behaviors. Though past clinical trials for osteoarthritis and depression were unsuccessful, emerging evidence supports DOR’s therapeutic potential in migraine, substance use disorder, irritable bowel syndrome, and metabolic disorder. Advances in pharmacology, including structure-guided design and intracellular targeting, offer promising new directions for DOR-based drug development.

## Introduction

The opioid receptor family is comprised of the mu opioid receptor (MOR), delta opioid receptor (DOR), and kappa opioid receptor (KOR), as well as the nociceptin receptor. In certain ways, the DOR is the forgotten middle child of this family. Unlike the MOR, it does not have spectacularly addictive properties or cause substantial respiratory depression; and unlike the KOR, the DOR does not enhance negative affective states, such as dysphoria. DOR-mediated responses appear to be solidly middle ground—restoring balance rather than evoking huge shifts in pain perception or mood. The DOR is a class A G-protein—coupled receptor (GPCR) that primarily couples to the inhibitory Gαi/o/z subunit [1]. The endogenous ligands for DOR are opioid peptides derived from the proopiomelanocortin (*POMC*), prodynorphin (*PDYN*), and proenkephalin (*PENK*) genes. While enkephalin peptides have a higher affinity for DORs over the other opioid receptors, all opioid peptides exhibit some degree of binding to the DOR [1]. The DOR is widely expressed in the central and peripheral nervous systems, as well as in the gut, pancreas, and lung. It is highly expressed in pain-processing regions including the spinal cord and dorsal root ganglia (DRG). There is also high expression of DORs in forebrain regions such as the cortex, caudate putamen, and olfactory bulb, as well as in the amygdala and hippocampus [2,3]. DOR expression is largely conserved between rodents and humans, although there are some differences. For example, the DOR is expressed throughout the mouse spinal cord but limited to superficial layers of the dorsal horn in humans.

**Figure 1:**
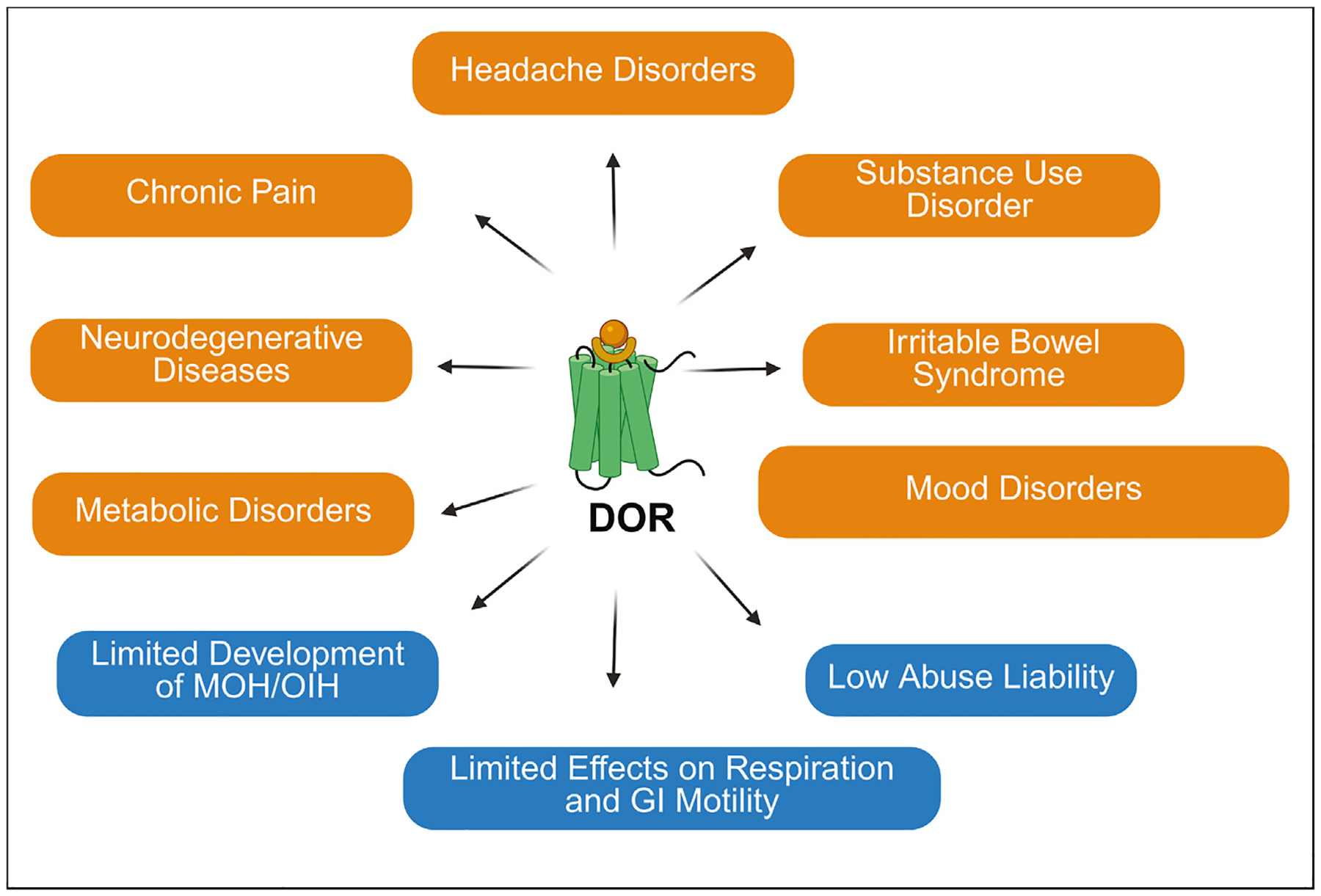
Therapeutic potential of the DOR. DOR, delta opioid receptor; MOH, medication-overuse headache; OIH, opioid-induced hyperalgesia; GI, gastrointestinal.

Unlike MOR agonists, DOR agonists show low efficacy in models of acute pain and are generally not anti-nociceptive. However, selective DOR agonists have demonstrated efficacy in alleviating chronic pain, including in animal models of inflammatory and neuropathic pain. It also shows potential in preventing ischemia, treating neurodegenerative diseases, addressing alcohol and opioid use disorders, and managing depression and anxiety disorders [4–6]. Beyond the central nervous system, DOR antagonists and positive allosteric modulators (PAMs) are under investigation for gastrointestinal disorders like irritable bowel syndrome (IBS) [7]. Importantly, DOR agonists have low abuse potential and produce minimal respiratory depression. Despite these characteristics, the clinical translation of the DOR has faced challenges, and no DOR-selective drug has yet received approval for human use. Clinical trials of DOR agonists, such as ADL5859 and ADL5747 for pain management, and AZD7268 and AZD2327 for major depressive disorder, did not meet primary end-points [8]. Although these results were disappointing, DOR agonists were generally well tolerated with few adverse effects. In addition, AZD2327 did significantly decrease the secondary endpoint of anxiety [9]. More recent studies have revealed a role for DORs in headache disorders, substance use disorders, and metabolic disorders which open new avenues for drug development of this receptor (Figure 1).

## DOR and headache disorders

The DOR has garnered significant interest as a novel therapeutic target for migraine and headache disorders, attributed to its extensive distribution in migraine-relevant regions and its unique pharmacological properties. The DOR is widely expressed in key regions involved in migraine pathophysiology, such as the trigeminal ganglia (TG) and trigeminal nucleus caudalis (TNC) [10], as well as the cortex. Preclinical studies demonstrated the effectiveness of DOR agonists in blocking cephalic allodynia induced by chronic migraine triggers like nitroglycerin, calcitonin gene—related peptide (CGRP), and pituitary adenylate cyclase—eactivating polypeptide (PACAP), as well as in models of migraine aura, post-traumatic headache, and medication-overuse headache (MOH) [8,**11,^12^]. Furthermore, compared to conventional therapies like sumatriptan and MOR-selective opioids, DOR agonists are associated with a reduced risk of MOH or opioid-induced hyperalgesia [12]. Recent studies have shown that enkephalinase inhibitors, such as PL37, enhance DOR-mediated effects, by preventing the degradation of enkephalins, thereby offering another pharmacological strategy for migraine treatment [13,14].

The mechanism through which DOR activation blocks migraine is under investigation. In the TNC and TG, the DOR is coexpressed with the endogenous migraine trigger CGRP and its receptor [10]. This coexpression suggests that DOR activation could inhibit CGRP release presynaptically, as well as CGRP receptor signaling postsynaptically, thereby contributing to its efficacy in alleviating headache symptoms. A more recent study investigated the role of DORs in PACA-Pergic signaling. PACAP is a known human migraine trigger and has been successfully targeted for migraine [15]. The vast majority of DOR-expressing neurons in the somatosensory cortex, hippocampus, and TNC coexpressed the PACAP receptor—pituitary adenylate cyclase—activating polypeptide type I (PAC1) [11]. Additionally, ~30–40% of DOR + cells in the somatosensory cortex and TNC coexpressed PACAP. Taken together, these results indicate that the DOR is well positioned in key anatomical and cellular regions to modulate molecular mechanisms of migraine. Ongoing research is focused on optimizing DOR agonist safety and efficacy while uncovering the precise circuits and signaling pathways that mediate their anti-migraine effects.

## Novel findings on DOR and pain

An emerging theme in the field is the action of endogenous opioids on DORs to facilitate recovery from chronic pain states. Mice with constitutive knockout of DORs took almost twice as long to recover from inflammatory pain compared to wildtype controls [16]. There is also consistent evidence to show that OPRD1 transcript levels in the DRG negatively correlate with allodynia (less expression) versus pain relief (increased expression) in a cisplatin model of chemotherapy-induced peripheral neuropathy (CIPN) [17,18]. An injection of the DOR antagonist, naltrindole, restored pain in mice that had recovered from CIPN [19], supporting the role of enkephalin—DOR tone in the masking of CIPN-induced chronic pain. Additionally, DORs in DRG neurons were critical for the pain-relieving effects of an HDAC6 inhibitor in this model [18]. These studies highlight the possibility that DORs may be pivotal in recovery from pain and that they may be a good target for CIPN.

Several recent studies show that DORs may bridge the immune system with pain processing. In one study, pain resolution in the CIPN model was regulated by tonic IL-10 release from resident macrophages in the meninges which upregulated DOR expression in peripheral sensory neurons [17]. In this case, the DOR was critical for the pain-remitting effects of IL-10, and conditional knockout of DOR in DRGs reinstated cisplatin-induced allodynia [17]. Furthermore, regulatory T cells (T_reg_) in the meninges surrounding the spinal cord were shown to release enkephalin which decreased allodynia by binding to DORs on MrgprD-expressing neurons in the DRG [*20]. Interestingly, this T cell—enkephalin—DOR restraining of nociception was sexually dimorphic and dependent on female sex hormones [20]. These studies reveal novel roles of the DOR in pain regulation, and boosting this endogenous action would be a valuable therapeutic strategy.

The endogenous regulation of pain processing by DORs goes beyond the peripheral nervous system. A recent study examined the circuits underlying placebo analgesia and identified DORs as critical to mediating this effect. This group developed a placebo analgesiaconditioning model to identify a circuit from the rostral anterior cingulate cortex (rACC) to the pontine nucleus, the activity of which was elevated when animals were in a location in which they expected pain relief. Neurons from the rACC project onto DOR-expressing cells in the pontine nucleus, which are critical for this placebo-induced analgesia [**21]. This is the first proper characterization of DOR cells in the brain pontine nucleus and reveals a novel role for these receptors in placebo analgesia and expectation of pain relief. This study also suggests that central nervous system—penetrant DOR agonists may be more efficacious pain therapies.

## DOR and convulsions

Despite the promising efficacy of DOR agonists, their development has been hindered by seizure liability [22]. Intriguingly, some DOR agonists induce convulsions, while others do not, and the underlying mechanisms remain under active investigation. DOR-agonist—induced convulsions are dependent on DORs and have the lowest efficacy requirement compared to other DOR-induced behaviors [**23]. DOR-mediated convulsions have been linked to GABAergic transmission in the forebrain, hippocampus, amygdala, and frontal cortex. DOR signaling targets G protein-coupled inwardly rectifying potassium channels (GIRK) or voltage-sensitive Ca^2+^ channels, leading to the disinhibition of pyramidal cells [22,24,25].

Ongoing efforts aim to develop DOR agonists without convulsive effects. One hypothesis is that DOR-agonist—induced convulsion is through the recruitment of β-arrestin 2. This has resulted in the development of several G-protein—biased ligands. A novel and selective DOR agonist, PN6047, is structurally similar to SNC80 but did not produce convulsions in rats at doses up to 80 mg/kg—well above the doses needed to produce DOR-mediated pain relieving and antidepressant-like effects [26]. This compound was reported by the company to have successfully completed phase 1 clinical trial. Another G-protein—biased DOR agonist, TRV250, completed phase 1 clinical trial and was well tolerated, warranting further investigation [27]. Several studies have examined the ligand—receptor complex to understand why certain DOR agonists induce convulsions. Agonists with a benzamide backbone, such as SNC80 and BW373U86, pose a high risk of seizure-like activity, whereas incorporating a piperidine substructure, as in PN6047, reduces this risk [22].

Another strategy to reduce adverse effects of DORs is the development of partial agonists which have a lower intrinsic efficacy at the receptor. Structure-guided design was used to rationally develop a novel selective DOR partial agonist (C6-Quino) which retained pain-relieving properties without proconvulsant effects [28]. The authors took advantage of the finding that the sodium-binding pocket on the DOR confers negative allosteric modulator activity [29] and undergoes significant conformational changes upon ligand binding [30]. C6-Quino significantly blocked allodynia in mouse models of neuropathic, inflammatory, and migraine pain. Notably, it did not induce convulsions or significantly alter respiration and locomotion at therapeutic doses [*28]. Additionally, a recent Cryo-electron microscopy (cryo-EM) structure of DORs bound to ADL5859 allowed for the structure-guided design of a novel G-protein—biased agonist, ADL06, which maintained efficacy in models of inflammatory, neuropathic, and visceral pain without seizurogenic properties. Furthermore, ADL06 also showed decreased effect on gut motility compared to first-generation DOR agonists or MOR agonists [31]. Virtual drug screening methods have also been used to identify novel ligands with fewer adverse effects. Screening a natural product library identified quinolizidine alkaloid cytisine derivatives as DOR agonists [32] which merit further characterization.

Several unresolved questions remain about why and how some DOR agonists can produce convulsions. First, it is unclear that the recruitment of β-arrestin 2 evokes proconvulsant effects as nonconvulsant agonists such as ADL5859 and ARM390 also recruit β-arrestin 2 [22,31].

Differential recruitment of G_α_ or G_βγ_ subunits, G protein-coupled receptor kinases (GRKs), or β-arrestin 1 may provide novel insights. Secondly, the molecular and cellular mechanisms and circuits underlying DOR-induced convulsions are largely unexplored. Convulsions are rapidly induced and terminated following DOR-agonist administration, suggesting activation of distinct signaling cascades versus genetic or epigenetic modulation. Future studies will hopefully shed light on how best to mechanistically avoid this adverse effect.

## Novel DOR signaling and pharmacology

The DOR is expressed both at the cell membrane and on intracellular pools. Stress, inflammation, pain, alcohol, and exposure to MOR agonists can enhance DOR trafficking to the cell surface [33–36]. Traditionally, GPCRs like DORs were thought to signal exclusively from the cell surface; however, recent research has demonstrated that DOR can continue to signal from intracellular compartments, including endosomes, after internalization [37]. The development of nanoparticles that deliver DOR agonists directly to these intracellular endosomes allowed researchers to prolong inhibition of pain-sensing neurons and provide extended pain relief [38]. This is an exciting area of research, and future studies on DOR and other GPCRs could reveal novel signaling and behavioral effects induced by activation of these intracellular receptor populations.

Efforts to increase or prolong endogenous opioid action through PAMs are ongoing. This strategy offers the advantage of enhancing the endogenous system without superactivation of the receptor. A novel DOR PAM was developed for the treatment of IBS [7]. DOR activation in the enteric nervous system can decrease gut motility but to a much lesser extent than MOR. This effect could be beneficial in IBS, and the DOR PAM, BMS-986187, appears to decrease colonic motility without causing constipation [7]. DOR PAMs could also be beneficial in treatment for chronic pain and migraine where there is a physiological upregulation of endogenous opioids and/or DORs. A challenge to this strategy is that although opioid peptides regulate neurotransmission [39,40], the mechanisms of endogenous opioid release and their subsequent behavioral effects remain poorly understood [41,42]. Advanced techniques, such as genetically encoded biosensors [43] and peptide-activated receptor fluorescence [44], offer promising insights into enkephalin-release intensity and location.

## Therapeutic potential for negative modulation of DORs

Pharmacological research on DORs has focused on enhancing DOR activity through agonists and PAMs. However, recent findings suggest that DOR activation under specific conditions could have adverse effects. Preclinically, early-life adversity can lead to reduced sensitivity to pain, accelerated morphine tolerance, and persistent inflammatory pain in adulthood. This effect appears to be dependent on increased DOR expression in the spinal cord [45]. This study highlights how little is known about DORs in development and aging. DORs may also play a facilitatory role in metabolic disorders. The DOR is highly expressed in the human pancreas, and in a clinical study, OPRD1 loss-of-function mutations increase adiposity while reducing hyperglycemia risk. In contrast, OPRD1 gain-of-function mutations were associated with lower adiposity and improved lipid metabolism but significantly increased the risk of type 2 diabetes. Additionally, the DOR antagonist naltrindole enhanced insulin secretion [**46]. We are still discovering novel roles for the DOR, and this study elucidates potential adverse consequences that may arise with chronic DOR activation. It also opens the possibility for the development of DOR-negative allosteric modulators [47] and antagonists.

## Conclusions

The DOR continues to hold promise as a therapeutic target for neuropsychiatric disorders. The idea of DOR agonists for the treatment of pain is challenged by the failed clinical trials in osteoarthritis, yet the DOR keeps emerging in pain studies, including as an endogenous mechanism for pain masking, in placebo-induced analgesia, or as a regulator of neuroimmune interactions. In addition, the DOR is a promising target for migraine and other headache disorders, which are mechanistically distinct from other chronic pain states. DORs may also be beneficial where there are comorbidities with pain and mood disorders, which is often the case in chronic pain/migraine. The DOR has also been identified for the treatment of alcohol or opioid use disorder and may be particularly beneficial in prolonged abstinence where individuals experience increased hypersensitivity as well as susceptibility to anxiety and depression. Novel pharmacological approaches help to solve the problem of proconvulsant effects of DOR agonists. Finally, novel roles of DORs in metabolic disorders could open up new therapeutic avenues for this receptor. While DORs may not be the most popular opioid receptor, it still has plenty of untapped potential.

## Nomenclature

CGRP: Calcitonin Gene-Related Peptide
DOR: Delta Opioid Receptor
GPCR: G protein-coupled receptor
IBS: Irritable Bowel Syndrome
KOR: Kappa Opioid Receptor
MOR: Mu Opioid Receptor
NAM: Negative Allosteric Modulator
NOP: Nociceptin
OPRDI: Gene encoding DOR
PAC1: Pituitary Adenylate Cyclase-Activating Polypeptide type I Receptor
PACAP: Pituitary Adenylate Cyclase Activating Polypeptide
PAM: Positive Allosteric Modulator
PDYN: Prodynorphin
PENK: Proenkephalin
POMC: Proopiomelanocortin
TG: Trigeminal Ganglia
TNC: Trigeminal Nucleus Caudalis
